# CD38 Ligation in Peripheral Blood Mononuclear Cells of Myeloma Patients Induces Release of Protumorigenic IL-6 and Impaired Secretion of IFN**γ** Cytokines and Proliferation

**DOI:** 10.1155/2013/564687

**Published:** 2013-12-30

**Authors:** Giorgio Fedele, Marco Di Girolamo, Umberto Recine, Raffaella Palazzo, Francesca Urbani, Alberto L. Horenstein, Fabio Malavasi, Clara Maria Ausiello

**Affiliations:** ^1^Department of Infectious, Parasitic and Immune-mediated Diseases, Anti-infectious Immunity Unit, Istituto Superiore di Sanità, 00161 Rome, Italy; ^2^Department of Internal Medicine S. Giovanni Calibita, Fatebenefratelli General Hospital, 00186 Rome, Italy; ^3^Onco-hematological unit, Department of General Medicine, S. Spirito Hospital, 00193 Rome, Italy; ^4^Laboratory of Immunogenetics, Department of Medical Sciences and Research Center for Experimental Medicine (CeRMS), University of Torino Medical School and “Città della Salute e della Scienza” Hospital, 10126 Torino, Italy

## Abstract

CD38, a surface receptor that controls signals in immunocompetent cells, is densely expressed by cells of multiple myeloma (MM). The immune system of MM patients appears as functionally impaired, with qualitative and quantitative defects in T cell immune responses. This work answers the issue whether CD38 plays a role in the impairment of T lymphocyte response. To this aim, we analyzed the signals implemented by monoclonal antibodies (mAb) ligation in peripheral blood mononuclear cells (PBMC) obtained from MM patients and compared to benign monoclonal gammopathy of undetermined significance (MGUS). PBMC from MM both failed to proliferate and secrete IFN*γ* induced by CD38 ligation while it retained the ability to respond to TCR/CD3. The impaired CD38-dependent proliferative response likely reflects an arrest in the progression of cell cycle, as indicated by the reduced expression of PCNA. CD38 signaling showed an enhanced ability to induce IL-6 secretion. PBMC from MM patients displays a deregulated response possibly due to defects of CD38 activation pathways and CD38 may be functionally involved in the progression of this pathology via the secretion of high levels of IL-6 that protects neoplastic cells from apoptosis.

## 1. **Introduction**

CD38 is a multifunctional surface molecule, expressed in a variety of cells and tissues. The molecule is densely expressed by normal plasma cells and by cells of multiple myeloma (MM), a clonal malignant disorder of terminally differentiated B lymphocytes. The disease is characterized by bone marrow plasmacytosis, bone lytic lesions, and by a secondary hypergammaglobulinemia. MM usually develops from an asymptomatic premalignant stage of clonal plasma cell proliferation, termed “monoclonal gammopathy of undetermined significance” (MGUS) [1, 2].

CD38 is simultaneously a receptor and adhesion molecule as well as an ectoenzyme that catalyses the synthesis of ADP ribose (ADPR), cyclic ADPR (cADPR), and nicotinic acid adenine dinucleotide phosphate (NAADP), starting from nicotinamide adenine dinucleotide (NAD^+^). cADPR and NAADP are two potent second messenger for Ca^2+^ release [3, 4]. As a receptor, CD38 is engaged by CD31, identified as a counter-receptor [5], or by surrogate agonistic monoclonal antibodies (mAbs) [6]. The effects mediated by CD38 ligation include production of pro-inflammatory and regulatory cytokines by monocytes [7], NK cells [8], activated B [9] and T lymphocytes [10] and dendritic cells (DC) [11], proliferation of T lymphocytes [12], and protection of mature B lymphocytes and DC from apoptosis [13, 14].

The role of CD38 has been informative in different pathological disorders, such as in AIDS (where CD38 is one of the earliest indicators of infection [15]) and B cell chronic lymphocytic leukemia (B-CLL) [16]. There are several issues suggesting that CD38 plays significant roles in MM. First, CD38 is expressed by normal and tumoral plasma cells at high levels, in cells which tend to eliminate the majority of surface molecules. Second, plasma cells from MM and MGUS express CD31, the CD38 ligand, in a significant proportion of cases [17–20]. Another finding linking CD38 and plasma cell biology is the release of interleukin (IL)-6 driven by CD38 signaling [7, 10]. Indeed, IL-6 produced by bone marrow stromal cells is an autocrine growth factor for human myeloma cells and it is involved in the genesis of several of the clinical symptoms observed in MM patients [20, 21]. However, still elusive is the functional role exerted by CD38 in plasma cells and in myeloma [2, 19].

The immune system of MM patients is functionally impaired, with quantitative and qualitative defects mainly in the context of cellular responses. Defects in antigen presenting cell (APC) functions have been reported in these patients. Indeed, high potency blood DC failed to up-regulate the expression of the costimulatory molecule CD80 in response to stimulation by human CD40 ligand, a defect caused by transforming growth factor *β*1 (TGF*β*1) and by (IL)-10 produced by malignant plasma cells [22]. DC functions were restored by inhibiting p38 or activating MEK/ERK MAPK and neutralizing IL-6 in progenitor cells [23]. Also, T lymphocytes from MM patients displayed an altered phenotype, characterized by enhanced expression of CD38 and HLA Class II [24], impaired responses to mitogens [25], and increased susceptibility to apoptosis (enhanced expression of Fas (APO-1/CD95) and decreased expression of the anti-apoptotic factor Bcl-2) [26, 27]. CD8^+^ T lymphocytes in MM patients are characterized by increased expression of nonfunctional killer inhibitory receptor CD94 [28].

Some of these defects may be linked to the superior ability of DC obtained from MM patients to maintain regulatory T cells [29]. Active MM patients show a deregulated cytokine network [30, 31], with increased synthesis, release of IL-6 production [25], and impaired Th1 response. The picture is further complicated by the finding of differences in Th1 response between active MM and MGUS patients as well as MM in remission [31].

Trying to dissect the role of CD38 in the immune response and at the same time to elucidate its role in MM pathogenesis, we designed a pilot study, where CD38-mediated signaling in MM and MGUS patients are compared. The rational stems from the notion that 25% of the patients with MGUS eventually develop MM or related plasma cell disorder. However, whether the changes that occur in the clonal plasma cell are important in the progression of MGUS to active myeloma is not yet clear. A critical feature shared by MM and MGUS is the extremely low rate of clonal plasma cell proliferation until late stages of MM are reached [32].

The approach adopted was a functional evaluation of CD38 engagement and signals in peripheral blood mononuclear cells (PBMC) obtained from MM patients and compared to a MGUS picture.

## 2. **Patients, Materials, and Methods**

### 2.1. Patients

PBMC were obtained from 11 patients with MM (mean age 74 years, 64% males) and 7 patients with MGUS (mean age 67 years, 71% males). Blood samples were taken before therapy. MM patients were staged according to the criteria of Durie and Salmon [33]. All stages were included: 4 patients of IA stage (36%), 3 patients of IIA stage (27%), and 4 patients of IIIA stage (36%). Fifteen healthy donors, sex and age matched were included as reference controls. All experiments were conducted in accordance with the Declaration of Helsinki [34]. Ethics approval was obtained from the Ethical Committee of two Hospitals, and written informed consent was provided by patients and healthy donors involved in the study.

### 2.2. Monoclonal Antibodies and Reagents

Agonistic anti-CD38 mAb [IB4 (IgG_2a_)] [35] and the reference anti-TCR/CD3 mAb [CBT3G (IgG_2a_)] were high-grade purified [36]. Proliferation tests and cytokine assays were performed by using the IB4 mAb at 20 *μ*g/mL, while CBT3G mAb was used at 1 *μ*g/mL.

### 2.3. PBMC Isolation and Proliferation and Cytokine Assays

PBMC obtained from heparinized venous peripheral blood by centrifugation on density gradient (Lympholyte-H, Cedarlane, Hornby, ON, Canada) were washed twice and suspended in RPMI-1640 medium (Gibco Life Technologies, Paisley, UK) supplemented with 5% pooled AB serum and antibiotics (Penicillin 50 IU/mL, Streptomycin 50 *μ*g/mL, Gibco) (hereinafter referred to as complete medium).

PBMC proliferation was measured by using 2 × 10^5^ cells/well in 0.2 mL complete medium in triplicate, in 96 flat-bottomed microwell trays (Falcon, BD Biosciences, San Jose, CA) in the presence of the relevant stimulus. The plates were incubated at 37°C in a 5% CO_2_ for 5 days. Eighteen hours before harvesting, the plates were pulsed with Methyl-^3^H-thymidine (0.5 *μ*Ci/well, specific activity: 2.5 Ci/mmole, GE Healthcare, Piscataway, NJ) and DNA synthesis was evaluated by counting ^3^H-thymidine incorporation [35]. Proliferation data were expressed as stimulation index (SI), defined as the ratio between the counts per minute of the stimulated cultures and the background value of unstimulated cultures.

Cytokine release was assessed by culturing PBMC in 5 mL tubes (Falcon) at a concentration of 2 × 10^6^ cells/mL in 0.5 mL of complete medium at 37°C in a 5% CO_2_ atmosphere. Supernatants were collected after 18 hours and used to measure IFN*γ* and IL-6. The enzyme immunoassay system adopted (Quantikine, R&D Systems, Inc., Minneapolis, MN) displayed a sensitivity of 3 pg/mL for IFN*γ*, 0.7 pg/mL for IL-6, respectively.

### 2.4. mRNA Cytokine Expression by TaqMan Real-Time Reverse Transcriptase-PCR Analysis

TaqMan Real-time Reverse Transcriptase-PCR (Life Technologies, Paisley, UK) analysis was used to measure cytokine mRNA expression. Total RNA was extracted from PBMC, and reverse transcription was carried out as previously described [35]. TaqMan assays were performed according to the manufacturer's instructions with an ABI 7700 thermocycler (Life Technologies). PCR was performed, by amplifying the target cDNA (IL-6 and PCNA transcripts) and with beta-actin cDNA as endogenous control. Specific primers and probes were obtained from Life Technologies. Data obtained were analyzed with PE Relative Quantification software (Life Technologies). Specific mRNA transcript levels were expressed as fold increase compared to basal condition [11].

### 2.5. Statistical Analyses

Statistical descriptive analyses were carried out using the SPSS Inc (Chicago, IL) statistical package. Differences between mean values were assessed by two-tailed Student's *t*-test. The association between proliferation and IL-6 secretion was measured by applying a linear regression model and by calculating the Pearson correlation coefficient. *P* < 0.05 was considered statistically significant.

## 3. **Results**

### 3.1. CD38-Mediated Signals in PBMC Purified from MM and MGUS Patients

CD38-mediated signals were comparatively evaluated in PBMC obtained from MM and MGUS patients, using healthy individuals as reference. The pathway driven by CD38 was also compared to the activation ruled by TCR/CD3. Previous [12, 35] and present studies (Table 1) indicate that CD38 ligation in PBMC by agonistic anti-CD38 mAb is followed by high levels of proliferation in healthy individuals. The same signals in PBMC obtained from MM and MGUS patients give a proliferation impact lower than that observed in controls (*P* < 0.01). The results indicate that the effect is specific for CD38 signals, while the cascade mediated by the TCR/CD3 pathway is unaffected (Table 1).

CD38 ligation by mAb in cultured PBMC induces multiple cytokines (including IFN*γ* and IL-6), some of them identical to those induced via TCR/CD3 activation [37]. Thus, IFN*γ* and IL-6 released after CD38 and CD3 activations were studied comparatively.

Due to the limited numbers of PBMC recovered from patients and to the decision to give priority to the proliferation measurement, cytokine assays were feasible in 10 out of 11 MM patients, 4 out of 7 MGUS patients, and 10 out of 15 healthy individuals.

Agonistic anti-CD38 mAb (and control anti-CD3 mAb) are able to induce release in PBMC from healthy individuals of high levels of IFN*γ* and IL-6. The results obtained in patients indicate that the ability to release IFN*γ* after CD38 engagement is decreased, while the IL-6 levels increased in MM (Table 2) and MGUS (data not shown) patients. Because of a high interdonor variability, expected when dealing with patients with different genetic background and immunological history, and the small sample of patients studied, the statistical significance was reached only when the IL-6 levels observed in CD38-activated PBMC of MM patients were compared to control group. The high IL-6 levels observed in untreated PBMC from MM patients are due to the high spontaneous IL-6 secretion observed in 4/10 MM patients tested and probably reflect the presence of malignant cells producing IL-6. After looking at other cytokines not directly associated with tumor progression (e.g., TNF*α* and IL-5), we observed that the production in untreated MM is almost overlapping with that scored by control PBMC (data not shown). These findings seem to confirm that IL-6 likely parallels the tumoral growth [20]. The levels of IFN*γ* and IL-6 release obtained after TCR/CD3 activation of PBMC were similar, irrespective of the source of PBMC (Table 2).

**Figure 1:**
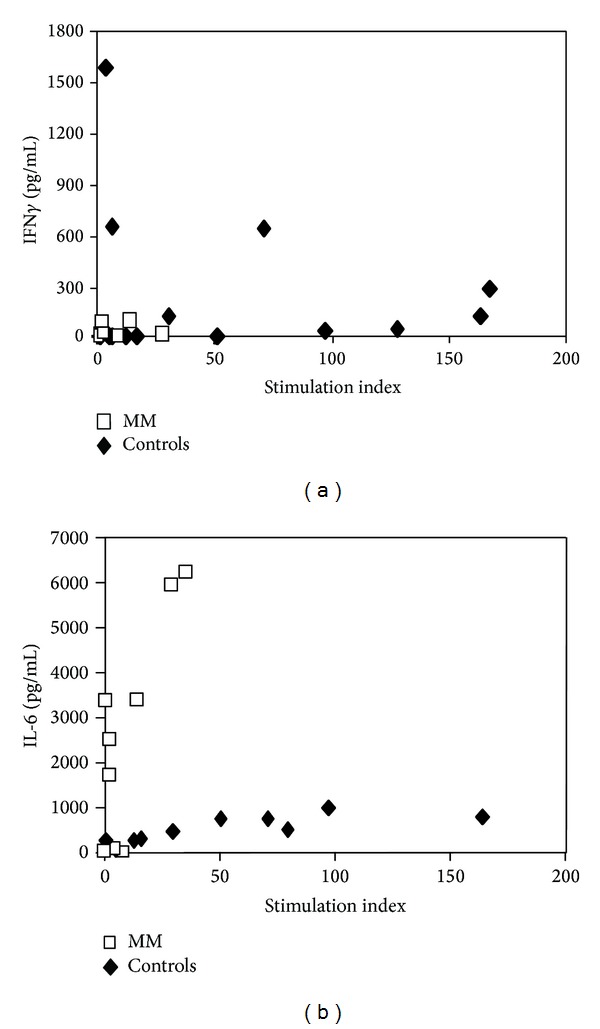
Proliferation, IFN*γ*, and IL-6 induction by CD38 mAb engagement in PBMC obtained from MM patients or healthy individuals. PBMC (2 × 10^5^ cells/well in 0.2 mL) obtained from MM patients or healthy controls were cultured in the presence of agonistic anti-CD38 IB4 (20 *μ*g/mL). Proliferation (SI) was measured after 5 days by ^3^H-Thymidine incorporation. IFN*γ* (pg/mL) and IL-6 (pg/mL) were measured by ELISA after 18 hours of culture. (a) Dispersion of proliferation (SI) respect IFN*γ* (pg/mL) values in each of all PBMC donors tested. (b) Dispersion of proliferation (SI) respect to IL-6 (pg/mL) values in each of all PBMC donors tested. For the definition of SI and technical details, see text.

Figures 1(a) and 1(b) show the IFN*γ* and IL-6 levels plotted with respect to lymphocyte proliferation levels detected after CD38 ligation in MM patients and in controls. The secretion of IFN*γ* is greatly reduced in PBMC from MM patients (Figure 1(a)); on the contrary, the secretion of IL-6 is significantly enhanced (Figure 1(b)), as compared to PBMC of control group. Figure 1(b) depicts a clear dichotomy between MM patients and healthy subjects in their response to CD38 stimulation. In PBMC of MM patients, the proliferation is almost absent while IL-6 production is elevated with a low correlation (*r* = 0.49, *P* = 0.014) between these two parameters. In healthy subjects, there is a clear correlation between PBMC proliferation and IL-6 levels (*r* = 0.65, *P* < 0.0001) with consistent levels of proliferation and reduced levels of IL-6 (Figure 1(b)). No correlation is found between IFN*γ* and proliferation (Figure 1(a)) both in patients and healthy subjects.

**Figure 2:**
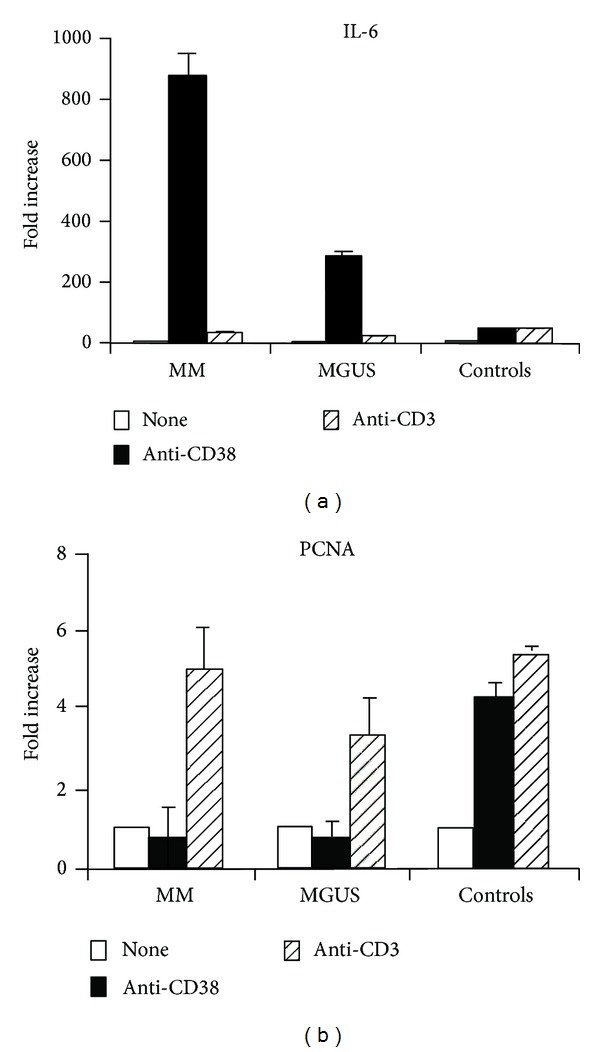
IL-6 and PCNA induction by CD38 molecules engagement in PBMC obtained from MM, MGUS patients, or healthy individuals. (a) PBMC (1 × 10^6^/mL) obtained from MM, MGUS patients, or healthy controls were cultured in the presence of agonistic anti-CD38, anti-CD3, or unstimulated (none). Taqman Real Time quantitative RT-PCR for IL-6 gene expression was performed at 18 hours time-point. mRNA transcript levels were expressed as fold increase over the unstimulated PBMC. (b) PBMC (1 × 10^6^/mL) obtained from MGUS, MM patients, or healthy individuals were cultured in the presence of agonistic anti-CD38, anti-CD3, or unstimulated (none). Taqman Real Time quantitative RT-PCR for PCNA gene expression was performed at 18 hours time-point. mRNA transcript levels were expressed as fold increase over the unstimulated PBMC. For further technical details, see text.

The increased levels of IL-6 induced by CD38 engagement in PBMC from MM and MGUS patients were confirmed at a transcriptional level. IL-6 mRNA expression measured by real-time quantitative RT-PCR highlights the same increase induced by CD38 triggering in MM and MGUS patients as compared to controls, paralleling the results of protein secretion (Figure 2(a))

### 3.2. The Impaired CD38-Dependent Proliferative Response in MM and MGUS Patients Is due to an Arrest in the Cell Cycle Progression

The finding that CD38 ligation by agonistic mAb was unable to induce proliferation in PBMC of MM and MGUS patients was hypothesized as due to a cell cycle arrest. To this aim, we tested the presence of proliferating cell nuclear antigen (PCNA), a cell marker used in MM patients to evaluate the proliferation of clonal plasma cells, and a parameter which also parallels the tumor histological grade [38]. PCNA expression was assessed by using real-time quantitative RT-PCR in CD38-activated PBMC. Results show that PCNA expression is not increased when PBMC from MM and MGUS are reacted with the agonistic anti-CD38 mAb, confirming the presence of a proliferative defect (Figure 2(b)). Conversely, PCNA expression is increased when the PBMC from MM and MGUS are exposed to an anti-CD3 mAb. This finding confirms that the ability of these cells to proliferate is maintained via TCR/CD3.

## 4. **Discussion**

The results obtained in this study support the view that PBMC from MM patients displays an altered response to signals mediated via CD38. Two lines of evidence indicate that CD38 is not a mere diagnostic marker but is also a key element in the pathogenetic events underlying myeloma development. First, the activation pathway ruled by CD38 appears as directly involved in the induction of a defective proliferative response in MM patients. Second, CD38 signals lead to the production of increased levels of IL-6, a key cytokine in the biology of MM [20, 21].

PBMC of MM and MGUS failed to proliferate after CD38 engagement, while the ability to proliferate in response to TCR/CD3 is maintained. This finding is suggestive of a specific defect in the CD38 activation pathways. The impaired CD38 proliferative response mediated by PBMC from MM and MGUS patients goes in parallel with an arrest in cell cycle progression, as indicated by the reduced expression of PCNA.

The experimental setting adopted does not allow to identify the cell population responsible for cytokine release in response to CD38 ligation. Yet, the cytokine data along with proliferation data (likely attributable to T cells) might highlight unresponsiveness to CD38 stimulation by T lymphocytes and provide support to the view that this pathway is deregulated in MM patients. However, these questions should be addresses more in depth by using multiparametric flow cytometry assays, allowing a better definition of the cells involved.

Myeloma cells may be directly involved in the inhibition of the T immune response of the patient [39, 40]. Campbell and colleagues [40] reported that myeloma cell lines suppress the proliferation of T lymphocytes, blocked in G1 arrest and refractory to respond to IL-2. Their conclusions are that MM cells have developed a strategy of suppression of T lymphocyte responses, which become unable to enter the IL-2/CD25 pathway of autocrine growth.

The lack of response to CD38 engagement by PBMC is not a general feature; indeed, CD38 ligation by agonistic mAb in PBMC obtained from MM leads to levels of IL-6 significantly increased as compared to the controls. Results in line with these are reported by Lapena and colleagues [25]. T lymphocytes from the MM patients activated by PHA release IL-6 in amounts significantly higher than those in the controls; at the same time, the proliferative response was decreased [25], in line with other results [31].

IL-6 is a major survival factor for malignant plasma cells *in vivo* and *in vitro*. Myeloma cell lines are protected from apoptosis by IL-6, due to the induction of specific antiapoptotic genes [41]. Besides, protecting from apoptosis, IL-6 plays a pivotal role in disease progression of MM patients. Indeed, autocrine IL-6 production detected in plasma cells from MM patients parallels a high labeling index [42].

This study reports that PBMC from MM and MGUS patients shares an impaired ability to transduce proliferative signals via CD38, suggesting that CD38 activation pathway defects are already present in the MGUS pathology and are not feature acquired by MM. A longitudinal study following the parameters here analyzed from the diagnoses of MGUS to the overt MM may help in detecting relevant defects in the above transition.

In conclusion, the present results support the role of CD38 in the genesis of tumor transformation of plasma cells. On one side, CD38 signaling pathways are able to rule IL-6 produced by PBMC, becoming a key step in the progression of the disease.

On the other side, it is apparent that myeloma induces a suppression of T cell responses, an event beneficial for tumor growth and survival. Myeloma is a tumor growing in closed system where the enzymatic activity of CD38 may be complemented by those exerted by PC-1/CD203a and CD73 [18, 43]. The consequence is that CD38 would be a component of one of the multiple strategies adopted by tumor to evade the immune response. This unconventional ectoenzyme network is able to provide the generation of local tolerance in different disease models, such as the BM microenvironment in pathology (e.g., myeloma or CLL) [44]. Conceivably, CD38/PC-1/CD73 pathway may tip the balance from activation to anergy and suppression [43, 45]. The fallouts of these observations are two-fold. One is to follow distribution and response to CD38 in the transition (if any) from MGUS to MM, and also to follow the proliferative response and IL-6 secretion in specific compartments of PBMC, such as T and B regulatory cells, DC subsets, NK-cells, and monocytes in order to link the alterations described to specific cell populations.

A second aspect concerns the applications in therapy. Targeted immunotherapy based on mAbs (murine, humanized, or human) specific for relevant tumor antigens has become a feasible and highly promising approach in hematological malignancies, mainly because it can be combined with conventional treatments to further increase the potency of antitumor effects. CD38 is a particularly attractive target on malignant plasma cells at all stages of disease and in CLL patients with a poor clinical prognosis or refractory to therapies. As such, this molecule is a promising target for antibody therapy also in different tumors [46].

## Figures and Tables

**Table 1:** Proliferation induced by CD38 ligation is specifically reduced in MM and MGUS patients.^a^

Stimulus	MM (*n* = 11)	MGUS (*n* = 7)	Controls (*n* = 15)
Anti-CD38	3.4 ± 1.2 *P* = 0.007	2.3 ± 0.8 *P* = 0.005	20.1 ± 5.3
Anti-CD3	36.6 ± 6.9	24.8 ± 10	44.0 ± 7.2
None	0.7 ± 0.1	0.4 ± 0.1	0.9 ± 0.4

^a^PBMC from MM, MGUS patients, and healthy individuals were treated with indicated stimulus and ^3^H-Thymidine incorporation assayed. Results are expressed as mean ± SE of cpm (×10^3^). Number (*n*) of individuals tested and statistical significance (Student's *t*-test) of differences between MM or MGUS patients versus healthy controls are indicated.

**Table 2:** Altered response in IFN*γ* and IL-6 cytokine release upon CD38 ligation in MM patients.^a^

Stimulus	MM		Controls	
	IFN*γ* (10)	IL-6 (10)	IFN*γ* (10)	IL-6 (10)
Anti-CD38	60 ± 19	2929 ± 878 *P* = 0.041	295 ± 156	820 ± 162
Anti-CD3	1072 ± 253	1638 ± 677	10361 ± 179	478 ± 99
None	45 ± 17	1088 ± 482	4.2 ± 1	236 ± 103

^a^PBMC from MM patients and healthy individuals were treated with indicated stimulus and IFN*γ* and IL-6 assayed by specific ELISA. Results are expressed as mean ± SE of pg/mL. Number (*n*) of individuals tested and statistical significance (Student's *t*-test) of differences between MM and healthy controls are indicated.

## References

[1] Kyle RA, Rajkumar SV (2008). Multiple myeloma. *Blood*.

[2] Kastrinakis NG, Gorgoulis VG, Foukas PG, Dimopoulos MA, Kittas C (2000). Molecular aspects of multiple myeloma. *Annals of Oncology*.

[3] Lee HC (2000). Enzymatic functions and structures of CD38 and homologs. *Chemical Immunology*.

[4] Malavasi F, Deaglio S, Funaro A (2008). Evolution and function of the ADP ribosyl cyclase/CD38 gene family in physiology and pathology. *Physiological Reviews*.

[5] Deaglio S, Morra M, Mallone R (1998). Human CD38 (ADP-ribosyl cyclase) is a counter-receptor of CD31, an Ig superfamily member. *Journal of Immunology*.

[6] Malavasi F, Deaglio S, Ferrero E (2006). CD38 and CD157 as receptors of the immune system: a bridge between innate and adaptive immunity. *Molecular Medicine*.

[7] Lande R, Urbani F, di Carlo B (2002). CD38 ligation plays a direct role in the induction of IL-1*β*, IL-6, and IL-10 secretion in resting human monocytes. *Cellular Immunology*.

[8] Mallone R, Funaro A, Zubiaur M (2001). Signaling through CD38 induces NK cell activation. *International Immunology*.

[9] Funaro A, Morra M, Calosso L, Zini MG, Ausiello CM, Malavasi F (1997). Role of the human CD38 molecule in B cell activation and proliferation. *Tissue Antigens*.

[10] Ausiello CM, la Sala A, Ramoni C, Urbani F, Funaro A, Malavasi F (1996). Secretion of IFN-*γ*, IL-6, granulocyte-macrophage colony-stimulating factor and IL-10 cytokines after activation of human purified T lymphocytes upon CD38 ligation. *Cellular Immunology*.

[11] Fedele G, Frasca L, Palazzo R, Ferrero E, Malavasi F, Ausiello CM (2004). CD38 is expressed on human mature monocyte-derived dendritic cells and is functionally involved in CD83 expressioin and IL-12 induction. *European Journal of Immunology*.

[12] Funaro A, Spagnoli GC, Ausiello CM (1990). Involvement of the multilineage CD38 molecule in a unique pathway of cell activation and proliferation. *Journal of Immunology*.

[13] Zupo S, Rugari E, Dono M, Taborelli G, Malavasi F, Ferrarini M (1994). CD38 signaling by agonistic monoclonal antibody prevents apoptosis of human germinal center B cells. *European Journal of Immunology*.

[14] Frasca L, Fedele G, Deaglio S (2006). CD38 orchestrates migration, survival, and Th1 immune response of human mature dendritic cells. *Blood*.

[15] Savarino A, Bottarel F, Malavasi F, Dianzani U (2000). Role of CD38 in HIV-1 infection: an epiphenomenon of T-cell activation or an active player in virus/host interactions?. *AIDS*.

[16] Deaglio S, Vaisitti T, Aydin S, Ferrero E, Malavasi F (2006). In-tandem insight from basic science combined with clinical research: CD38 as both marker and key component of the pathogenetic network underlying chronic lymphocytic leukemia. *Blood*.

[17] Chillemi A, Zaccarello G, Quarona V (2013). Anti-CD38 antibody therapy: windows of opportunity yielded by the functional characteristics of the target molecule. *Molecular Medicine*.

[18] Quarona V, Zaccarello G, Chillemi A (2013). CD38 and CD157: a long journey from activation markers to multifunctional molecules. *Cytometry B*.

[19] Chillemi A, Zaccarello G, Quarona V (2014). CD38 and bone marrow microenvironment. *Frontiers in Bioscience*.

[20] Gadó K, Domján G, Hegyesi H, Falus A (2000). Role of interleukin-6 in the pathogenesis of multiple myeloma. *Cell Biology International*.

[21] Bommert K, Bargou RC, Stühmer T (2006). Signalling and survival pathways in multiple myeloma. *European Journal of Cancer*.

[22] Brown RD, Pope B, Murray A (2001). Dendritic cells from patients with myeloma are numerically normal but functionally defective as they fail to up-regulate CD80 (B7-1) expression after huCD40LT stimulation because of inhibition by transforming growth factor-*β*1 and interleukin-10. *Blood*.

[23] Wang S, Hong S, Yang J (2006). Optimizing immunotherapy in multiple myeloma: restoring the function of patients’ monocyte-derived dendritic cells by inhibiting p38 or activating MEK/ERK MAPK and neutralizing interleukin-6 in progenitor cells. *Blood*.

[24] Massaia M, Bianchi A, Attisano C (1991). Detection of hyperreactive T cells in multiple myeloma by multivalent cross-linking of the CD3/TCR complex. *Blood*.

[25] Lapena P, Prieto A, Garcia-Suarez J (1996). Increased production of interleukin-6 by T lymphocytes from patients with multiple myeloma. *Experimental Hematology*.

[26] Frassanito MA, Silvestris F, Silvestris N (1998). Fas/Fas ligand (FasL)-deregulated apoptosis and IL-6 insensitivity in highly malignant myeloma cells. *Clinical and Experimental Immunology*.

[27] Massaia M, Borrione P, Attisano C (1995). Dysregulated Fas and bcl-2 expression leading to enhanced apoptosis in T cells of multiple myeloma patients. *Blood*.

[28] Besostri B, Beggiato E, Bianchi A (2000). Increased expression of non-functional killer inhibitory receptor CD94 in CD8+ cells of myeloma patients. *British Journal of Haematology*.

[29] Banerjee DK, Dhodapkar MV, Matayeva E, Steinman RM, Dhodapkar KM (2006). Expansion of FOXP3high regulatory T cells by human dendritic cells (DCs) in vitro and after injection of cytokine-matured DCs in myeloma patients. *Blood*.

[30] Murakami H, Ogawara H, Hiroshi H (2004). Th1/Th2 cells in patients with multiple myeloma. *Hematology*.

[31] Frassanito MA, Cusmai A, Dammacco F (2001). Deregulated cytokine network and defective Th1 immune response in multiple myeloma. *Clinical and Experimental Immunology*.

[32] Rajkumar SV, Fonseca R, Dewald GW (1999). Cytogenetic abnormalities correlate with the plasma cell labeling index and extent of bone marrow involvement in myeloma. *Cancer Genetics and Cytogenetics*.

[33] Durie BGM, Salmon SE (1975). A clinical staging system for multiple myeloma: correlation of measured myeloma cell mass with presenting clinical features, response to treatment, and survival. *Cancer*.

[34] World Medical Association (2013). World Medical Association Declaration of Helsinki: ethical principles for medical research involving human subjects. *Journal of the American Medical Association*.

[35] Ausiello CM, Urbani F, Lande R (2000). Functional topography of discrete domains of human CD38. *Tissue Antigens*.

[36] Horenstein AL, Crivellin F, Funaro A, Said M, Malavasi F (2003). Design and scaleup of downstream processing of monoclonal antibodies for cancer therapy: from research to clinical proof of principle. *Journal of Immunological Methods*.

[37] Ausiello CM, Urbani F, la Sala A, Funaro A, Malavasi F (1995). CD38 ligation induces discrete cytokine mRNA expression in human cultured lymphocytes. *European Journal of Immunology*.

[38] Chaidos AI, Bai MC, Kamina SA, Kanavaros PE, Agnantis NJ, Bourantas KL (2002). Incidence of apoptosis and cell proliferation in multiple myeloma: correlation with bcl-2 protein expression and serum levels of interleukin-6 (IL-6) and soluble IL-6 receptor. *European Journal of Haematology*.

[39] Cook G, Campbell JDM, Carr CE, Boyd KS, Franklin IM (1999). Transforming growth factor *β* from multiple myeloma cells inhibits proliferation and IL-2 responsiveness in T lymphocytes. *Journal of Leukocyte Biology*.

[40] Campbell JDM, Cook G, Robertson SE (2001). Suppression of IL-2-induced T cell proliferation and phosphorylation of STAT3 and STAT5 by tumor-derived TGF*β* is reversed by IL-15. *Journal of Immunology*.

[41] Jourdan M, Veyrune J-L, de Vos J, Redal N, Couderc G, Klein B (2003). A major role for Mcl-1 antiapoptotic protein in the IL-6-induced survival of human myeloma cells. *Oncogene*.

[42] Donovan KA, Lacy MQ, Kline MP (1998). Contrast in cytokine expression between patients with monoclonal gammopathy of undetermined significance or multiple myeloma. *Leukemia*.

[43] Horenstein AL, Chillemi A, Zaccarello G (2013). CD38/CD203a/CD73 ectoenzymatic pathway independent from CD39 runs a novel adenosinergic loop in human T lymphocytes. *OncoImmunology*.

[44] Malavasi F, Deaglio S, Damle R, Cutrona G, Ferrarini M, Chiorazzi N (2011). CD38 and chronic lymphocytic leukemia: a decade later. *Blood*.

[45] Bahri R, Bollinger A, Bollinger T, Orinska Z, Bulfone-Paus S (2012). Ectonucleotidase CD38 demarcates regulatory, memory-like CD8+ T cells with IFN-g-mediated suppressor activities. *PLoS ONE*.

[46] Ellis JH, Barber KA, Tutt A (1995). Engineered anti-CD38 monoclonal antibodies for immunotherapy of multiple myeloma. *Journal of Immunology*.

